# Lessons in the Diagnosis and Treatment of Eye Diseases

**Published:** 1892-06

**Authors:** 


LESSONS IN THE DIAGNOSIS AND TREATMENT OF EYE DISEASES. By CASEY A. WOOD, M. D. Physicians' Leisure Library. Price, cloth, 50 cents; paper, 25 cents. Detroit: Geo. S. Davis. 1891.

The author of this little manual states in the preface that it is intended "to aid the physician to detect and treat those diseases of the eye which experience has shown are most frequently overlooked in the course of general practice." Yet the subjects touched upon in the various chapters include nearly every affection of the eye, besides various operations, some of which are far too technical for the general practitioner. The style of the book is clear, and the arrangement of the matter good. E. S.

